# BMI1 promotes spermatogonial stem cell maintenance by epigenetically repressing Wnt10b/β-catenin signaling

**DOI:** 10.7150/ijbs.70441

**Published:** 2022-04-04

**Authors:** Jun Yu, Cong Shen, Meng Lin, Xia Chen, Xiuliang Dai, Zhiran Li, Yunhao Wu, Yangbo Fu, Jinxing Lv, Xiaoyan Huang, Bo Zheng, Fei Sun

**Affiliations:** 1 Institute of Reproductive Medicine, School of Medicine, Nantong University, Nantong 226001, China.; 2 State Key Laboratory of Reproductive Medicine, Center for Reproduction and Genetics, Suzhou Municipal Hospital, the Affiliated Suzhou Hospital of Nanjing Medical University, Gusu School, Nanjing Medical University, Suzhou 215002, China.; 3 State Key Laboratory of Reproductive Medicine, Department of Histology and Embryology, Nanjing Medical University, Nanjing 211166, China.; 4 Department of Obstetrics and Gynecology, Affiliated Hospital 2 of Nantong University and First People's Hospital of Nantong City, Nantong 226001, China.; 5 Center of Clinical Reproductive Medicine, The Affiliated Changzhou Maternity and Child Health Care Hospital of Nanjing Medical University, Changzhou 213000, China.; 6 Suzhou Dushu Lake Hospital (Dushu Lake Hospital Affiliated to Soochow University), Suzhou 215124, China.; 7 NHC Key Laboratory of Study on Abnormal Gametes and Reproductive Tract (Anhui Medical University), Hefei 230032, China.

**Keywords:** Spermatogonial stem cells, Self-renewal, BMI1, Wnt10b, Wnt/β-catenin signaling

## Abstract

The self-renewal of spermatogonial stem cells (SSCs) requires a special microenvironment and is strictly controlled. Previously, we identified BMI1 as a key regulator of spermatogenesis in a knock-out mouse model. However, the mechanisms by which BMI1 regulates SSC maintenance remain largely unknown. Herein, we show that BMI1 is essential for SSC maintenance. BMI1 directs the transcriptional repression of target genes by increasing H2AK119ub and reducing H3K4me3 in SSCs. Furthermore, BMI1 inhibition resulted in the transcriptional activation of *Wnt10b* and thereby promoted the nuclear translocation of β-catenin in SSCs. Importantly, the suppression of Wnt/β-catenin signaling restored both the cytoplasmic expression of β-catenin and SSC maintenance in BMI1-deficient SSCs. Finally, we demonstrated that Wnt/β-catenin signaling was also involved in BMI1-mediated SSC maintenance *in vivo*. Altogether, our study not only reveals a novel mechanism for BMI1 in the process of SSC maintenance, but also provides a potential new strategy for treating male infertility.

## Introduction

Spermatogonial stem cells (SSCs) originate from a population of the most primitive spermatogonia, which have the capacity for self-renewal and differentiation, and can maintain spermatogenesis throughout a man's lifetime 1. A stem cell niche is formed by somatic cell populations and SSCs within the testis, contributing to the SSC niche 2. The SSC microenvironment is precisely regulated by intrinsic and extrinsic signals 3. Several key factors such as Pou5f1 (Oct-3/4), Neurogenin 3 (Ngn3), Nanos homolog 2 (Nanos2), Paired box protein Pax-7 (Pax7), telomerase reverse transcriptase (Tert), and inhibitor of DNA binding 4 (Id4), are predominantly expressed in undifferentiated spermatogonia and play critical roles in SSC maintenance 4. However, the extent of SSC properties remains largely unknown.

BMI1 is a component of the Polycomb repressive complex 1 (PRC1), whose function is to epigenetically repress targeted genes 5. It has been identified as an oncogene which is required for the maintenance of stem cells and carcinoma formation 6-9. *Bmi1* knock-out mice showed impaired mitochondrial function and excessive reactive oxygen species (ROS) production, which resulted in defective hematopoietic cell self-renewal, premature ageing phenotypes, and a shortened lifespan 7, 10-13. In our previous study, we found that BMI1 was abundantly expressed in murine testes, and that the immunostaining of BMI1 in testicular sections revealed that it was widely distributed in all types of testicular cells, including germ and somatic cells 14. Furthermore, *Bmi1* knock-out mice exhibited impaired spermatogenesis, reduced serum testosterone levels, and male infertility 14. Recently, we have identified BMI1 both as an antioxidant factor and an epigenetic inhibitor in a mouse MLTC-1 Leydig cell line and primary Leydig cells 15, 16. BMI1 orchestrated steroidogenesis by maintaining redox homeostasis and epigenetically repressing p38 MAPK signaling 15, 16. Additionally, a study showed that the repression of endogenous BMI1 attenuated the proliferation of spermatogonia, while BMI1 overexpression exhibited the opposite effect in cultured spermatogonia *in vitro* 17. Furthermore, in a more recent study, we revealed that BMI1 is required for the proliferation of the mouse spermatogonia cell line GC-1 via the epigenetic repression of *Ptprm*, the gene encoding Protein tyrosine phosphatase receptor type M (PTPRM) 18. However, the definitive regulatory mechanism employed by BMI1 in SSCs remains largely unclear.

The Wnt signaling pathway is largely comprised of the canonical Wnt/β-catenin pathway and the noncanonical polarity pathway 19, 20. The dysfunction of Wnt/β-catenin signaling has been implicated in multiple developmental defects and major diseases 21, due to its involvement in major processes such as embryonic axis formation, sex determination, cell proliferation, differentiation, apoptosis, cell migration and fate division, thus maintaining a tight control over cell-cell communications 22, 23. Wnt ligands are recruited to bind a large oligomerized Frizzled-LRP5/6 complex and activate canonical Wnt/β-catenin signaling, inducing the sequestration of β-catenin inhibitors and therefore stabilizing β-catenin in the cytoplasm 24-26. Consequently, the blockade of β-catenin-specific phosphorylation and degradation, causes β-catenin to accumulate in the cytoplasm and translocate into the nucleus 27. The nuclear translocation of β-catenin leads to the formation of a transcriptional complex with T-cell factor/Lymphoid enhancer factor (TCF/LEF) on Wnt Response Elements (WREs), which subsequently regulates the expression of target genes, the nature of which varies according to the cellular context 24, 25, 27. In the last decade, Wnt signaling has also been suggested to be implicated in male germline maintenance; for instance, Wnt6 and Wnt3a were reported to promote SSC or progenitor cells proliferation via Wnt/β-catenin signaling 28, 29. Another study reported that Wnt5a supports SSCs self-renewal through the noncanonical Wnt pathway 30.

In this study, we predominantly focused on the role and regulatory mechanism of BMI1 in SSC maintenance, and demonstrate that BMI1 is essential for SSC maintenance by epigenetically repressing *Wnt10b* expression and the subsequent nuclear translocation of β-catenin both *in vitro* and *in vivo*. Our data strongly indicate that BMI1 is a crucial epigenetic mediator in SSC maintenance, and provide new targets for the treatment of male infertility.

## Materials and Methods

### Mice

*Bmi1* mutant mice were maintained in a specific-pathogen-free (SPF) grade experimental animal center of Nanjing Medical University. *Bmi1* heterozygous (+/-) mice were intercrossed to obtain *Bmi1* homozygous (KO; -/-) mice, which were genotyped by PCR amplification, as previously described 14. Animal use was approved by the Animal Ethical and Welfare Committee of Nanjing Medical University.

### Cell culture

The establishment of long-term *in vitro* murine SSC cultures was previously described 31. In short, SSCs were maintained with STO feeder cells in StemPro-34-based medium (Invitrogen, Carlsbad, CA, USA). Growth factors GDNF (R&D Systems, Minneapolis, MN, USA), EGF (Millipore Corporation, Billerica, MA, USA), and bFGF (R&D Systems) were used at the concentration of 15 ng/mL, 20 ng/mL, and 10 ng/mL, respectively. Culture medium was changed every 2 days and the SSC colonies were subcultured with new feeder cells every week. All experiments were performed using SSC colonies obtained after 5 passages. For short-term feeder-free cultures, plates were pretreated with Matrigel (BD Bioscience, San Jose, CA, USA) overnight at 4 °C. SSCs were maintained in a 37 °C humidified incubator containing 5% CO_2_.

### Immunofluorescence

The immunofluorescence of SSCs was conducted as previously described 32, 33. Briefly, SSCs were fixed in 4% (*w*/*v*) paraformaldehyde (PFA) for 15 minutes, blocked with 1% (*w*/*v*) bovine serum albumin (BSA, Sigma, St. Louis, MO, USA) for 2 hours at room temperature, and then labeled with the indicated primary antibodies (Table S1) overnight at 4 °C. Samples were subsequently probed with Alexa-Fluor-conjugated secondary antibodies (Thermo Scientific, Waltham, USA) for 2 hours at room temperature and stained with 4',6-diamidino-2-phenylindole (DAPI). For immunostaining of testicular sections, testes were fixed in 4% (*w*/*v*) PFA, dehydrated, embedded in paraffin, and cut into 5 μm sections. Sections were deparaffinized, rehydrated, and further labeled with the indicated primary antibodies (Table S1). Images were captured on an LSM810 confocal laser microscope (Carl Zeiss, Oberkochen, Germany).

### Western blotting

SSCs were lysed with a RIPA lysis buffer (Beyotime Institute of Biotechnology, Nantong, China), and the protein concentrations were assayed using a BCA kit (Beyotime Institute of Biotechnology) according to the manufacturer's instructions. Western blotting analysis was performed according to a routine protocol as previously reported 20. The primary antibodies used here are summarized in Table S1. Band signals were analyzed using Image-Pro Plus Software (Media Cybernetics, San Diego, CA, USA).

### Cell viability and apoptosis assays

Cell Counting Kit-8 (CCK-8) (Beyotime Institute of Biotechnology) was used to measure SSCs viability, according to the manufacturer's instructions. Shortly, SSCs were seeded into a feeder-free 96-well plate at 2,000 cells/well. The time point of cell seeding was identified as 0 hours. For each time point, the CCK-8 solution was added to the wells at a dilution of 1:200 and incubated for 2 hours. Thereafter, the absorbance at 450 nm was assayed using a microplate reader (EPOCH2, BioTec, Winooski,VT, USA).

Cellular apoptosis was detected using a Terminal deoxynucleotidyl transferase-dUTP nick-end labeling (TUNEL) kit (Vazyme, Nanjing, China), according to the manufacturer's instructions. Apoptotic signals were observed using an LSM810 confocal laser microscope (Carl Zeiss).

### Flow cytometry

SSCs were harvested and separated from feeder cells by gentle pipetting, reaching > 90% purity 30, 34. SSC colonies were trypsinized into single cells and fixed with 70% cold ethanol overnight at 4 °C. The single cell suspension was washed with phosphate-buffered saline (PBS) and stained with 40 μg/mL propidium iodide (PI) for half an hour at room temperature. Cell cycle dynamics were analyzed on a flow cytometer (BD Biosciences, Franklin Lakes, NJ, USA). SSCs were clearly distinguished from feeder cells by their low side scatter and homogeneous size 30, which further eliminated feeder cell contamination from the gated SSC population. For each sample, at least 10,000 events were collected.

### RNA extraction and quantitative real-time PCR

Total RNA was extracted using the TRIzol reagent (Invitrogen), and subsequently reverse transcribed into cDNA using a HiScript III 1st Strand cDNA Synthesis Kit (Vazyme), according to the manufacturer's instructions. PCR reactions were run on an ABI 7500 system (Applied Biosystems, Foster City, CA, USA), using a SYBR green qPCR MasterMix (Vazyme). 18S rRNA was used as a reference control. The primers used for quantitative real-time PCR are listed in Table S2.

### Chromatin immunoprecipitation (ChIP)

The ChIP assay was carried out using an EZ-Magna ChIP Kit (Millipore), as previously described 16, 18. SSCs or testicular tissues were trypsinized into single cells and cross-linked with 1% formaldehyde for 10 minutes at room temperature. Thereafter, the cell lysates were sonicated to shear cross-linked DNA into ~200-1000 bp pieces, and the chromatin fragments were incubated with the indicated antibodies (Table S1) overnight at 4 °C. Nearly 5% of the starting supernatant was removed as “Input”. Precipitated DNA was purified using spin columns and further subjected to quantitative real-time PCR analysis. Primers for ChIP-qPCR are listed in Table S3. The murine *Gapdh* (glyceraldehyde-3-phosphate dehydrogenase) gene was used as a negative control.

### Statistical analysis

GraphPad Prism 8 software was used for statistical analysis. *P*-values were calculated using a Student's *t*-test or one-way ANOVA followed by post-hoc Dunnett test with * *P* < 0.05; **P < 0.01; ***P < 0.001 denoting significance.

## Results

### BMI1 regulates SSC maintenance

**Figure 1:**
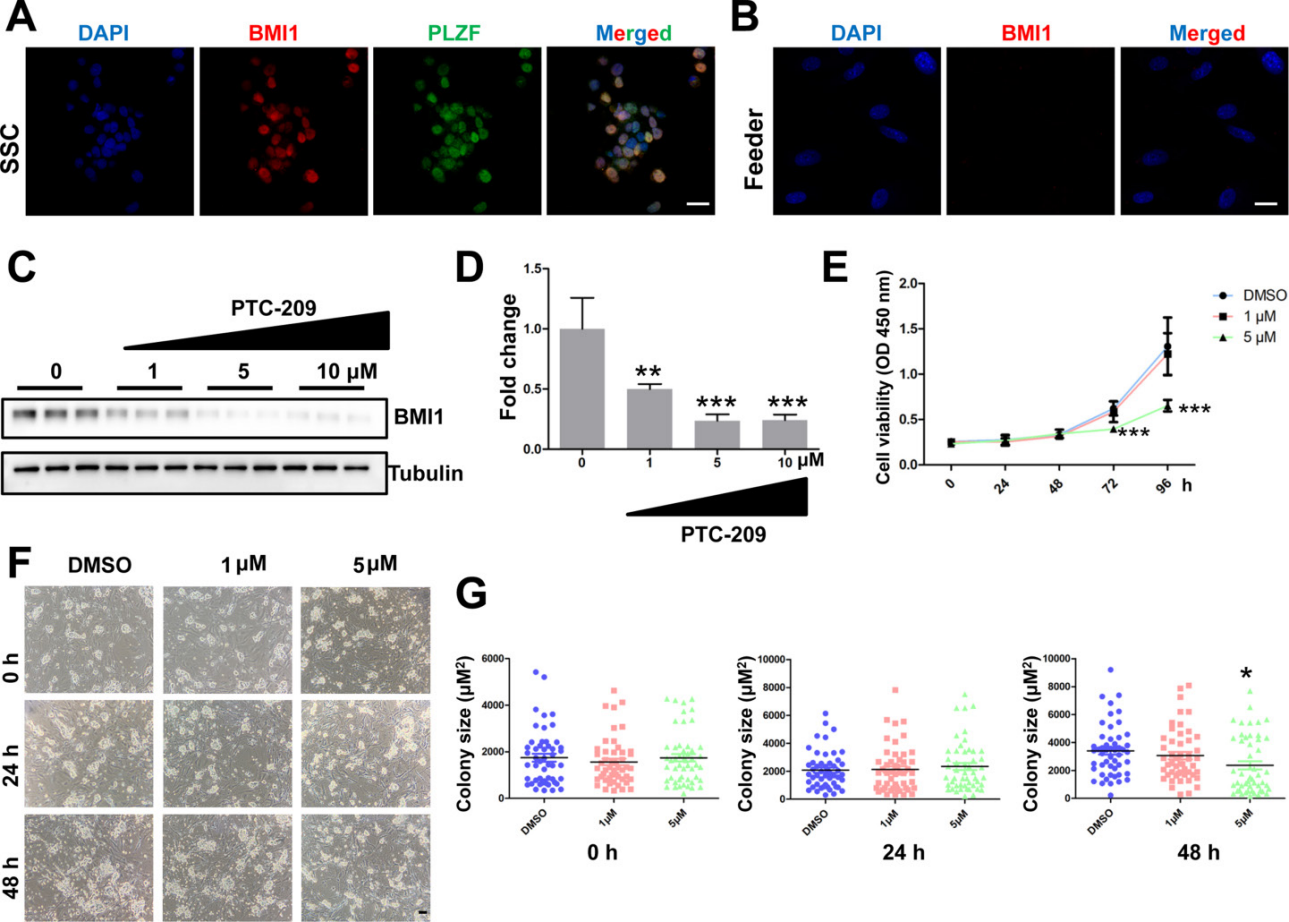
**Expression and function of BMI1 in cultured mouse SSCs. (A)** Co-immunostaining for BMI1 and PLZF in SSCs. Scale bar, 20 µm. The experiments were repeated three times. **(B)** Immunostaining of BMI1 in (STO) feeder cells. Scale bar, 20 µm. The experiments were repeated three times.** (C)** SSCs were treated with the indicated doses of PTC-209 for 48 h, and then removed from the feeder cells by gentle pipetting. Cells were lysed and subjected for western blotting analysis. **(D)** Quantification of (C). Data are presented as mean ± SD, n = 3 independently differentiated groups. **(E)** SSCs were treated with the indicated doses of PTC-209 for 48 h before being separated from feeder cells by gentle pipetting. Cells were then seeded into a feeder-free 96-well plate and subjected to the CCK-8 assay at the indicated time points. Data are presented as mean ± SD, n = 6 independently differentiated groups. **(F)** SSCs were treated with the indicated doses of PTC-209 for 48 h, and then assessed for colony size statistics at the indicated time points. After treatment with PTC-209 for 48 h, the culture medium was changed (labelled at the 0 h time point). Scale bar, 50 µm. **(G)** Quantification of SSC colony size of (F). Data represent mean ± SD, n = 50 colonies from three independently differentiated groups. For (D), (E), and (G), statistical analysis was performed using One-way ANOVA with Dunnett post hoc test. *P < 0.05; **P < 0.01; ***P < 0.001.

To test whether *Bmi1* was involved in controlling SSC maintenance, we established an *in vitro* murine SSC culture system, in which SSCs were cultured with feeder cells and growth factors, as previously described 31. We first used immunostaining to analyze BMI1 subcellular localization, and found that BMI1 was expressed in the nucleus of cultured SSCs (Figure 1A), but not in the feeder cells (Figure 1B). We next manipulated BMI1 levels in SSCs using a specific inhibitor, PTC-209 (Selleck, Houston, TX, USA) 35, 36. To determine the inhibitory efficiency of PTC-209, SSCs were treated with three concentrations (1, 5, and 10 μM) of PTC-209, according to our experience from a mouse spermatogonia cell line (GC-1) 18. Western blotting analysis showed that PTC-209 could substantially suppress BMI1 expression in a dose-dependent manner (Figure 1C and 1D). Moreover, we used the CCK-8 assay to investigate SSC viability after BMI1 inhibition. The growth of SCCs was dramatically inhibited after 72 hours of treatment with 5 μM PTC-209, whereas 1 μM PTC-209 had no measurable effect on SSC viability (Figure 1E). Meanwhile, we also found that SSC colony size was significantly reduced in 5 μM PTC-209-treated SSCs, but not in those treated with 1 μM PTC-209, 48 hours after inhibitor administration (Figure 1F and 1G). Since BMI1 was only expressed in SSCs, but not in feeder cells, we concluded that the inhibition of SSC growth was directly attributed to the action of BMI1 on SSCs.

### PTC-209 disturbs the balance between SSC proliferation and apoptosis

**Figure 2:**
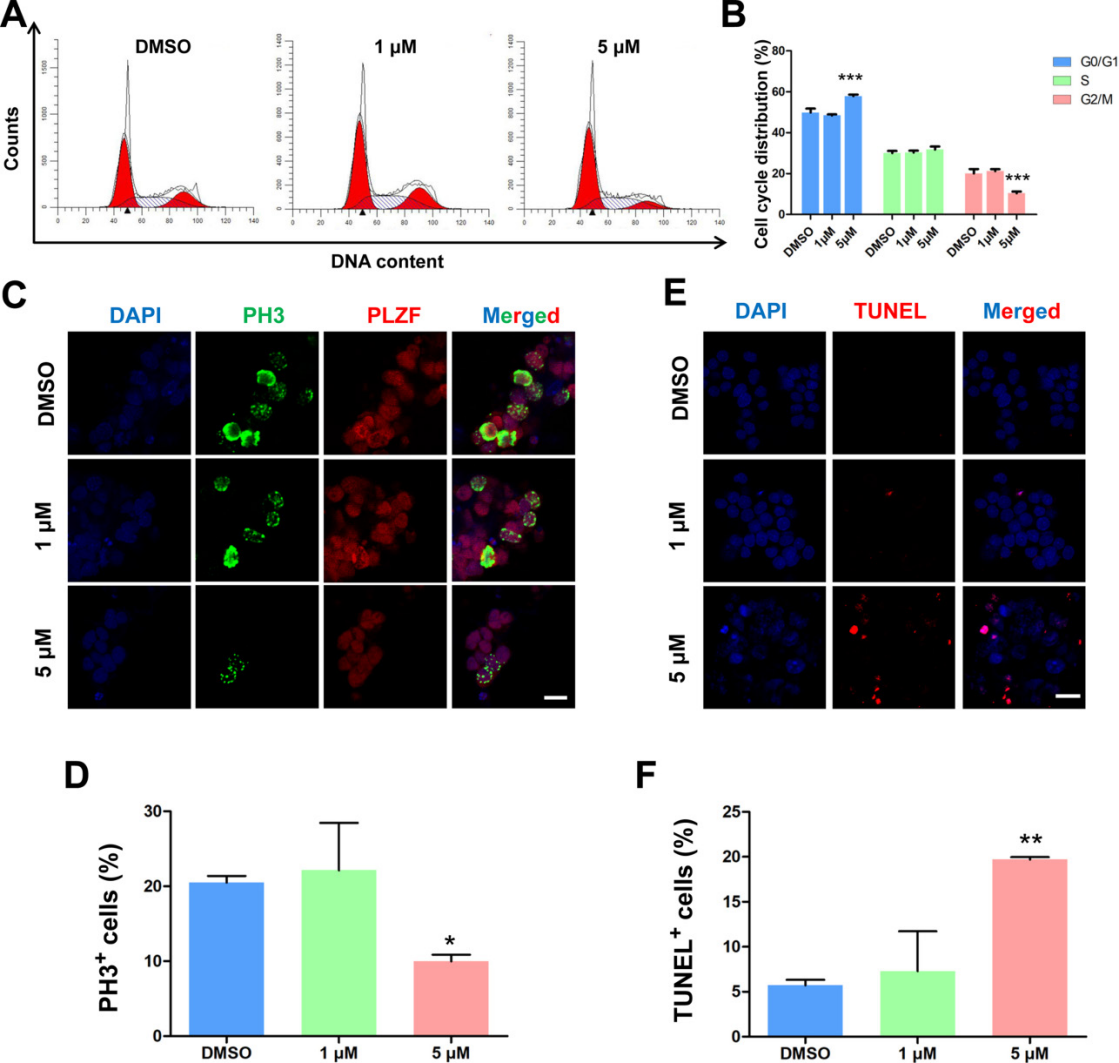
**Effects of PTC-209 on the cell cycle dynamics, cell proliferation, and apoptosis of SSCs. (A)** Flow cytometry histograms showing the extent of propidium iodide (PI) fluorescence on SSCs treated with DMSO, 1 µM, or 5 µM PTC-209 for 48 h. **(B)** Quantification of cell cycle distribution of (A). **(C)** Co-immunostaining for PH3 and PLZF in SSCs treated with DMSO, 1 µM, or 5 µM PTC-209 for 48 h. Scale bar, 20 µm.** (D)** Quantification of (C). **(E)** TUNEL staining of SSCs treated with DMSO, 1 µM, or 5 µM PTC-209 for 48 h. Scale bar, 20 µm.** (F)** Quantification of (E). For (B), (D), and (F), data represent mean ± SD, n = 3 independently differentiated groups. Statistical analysis was performed using One-way ANOVA with Dunnett post hoc test. *P < 0.05; **P < 0.01; ***P < 0.001.

To further investigate the roles of BMI1 in SSC maintenance, we examined the cell cycle dynamics of SSCs by flow cytometry after they were treated for 48 hours with PTC-209. After treatment with 5 μM PTC-209, BMI1-deficient SSCs were arrested at G0/G1 phase, with a marked decline in cells in the G2/M phase and an unaltered S phase distribution (Figure 2A and 2B). Moreover, the immunostaining of SSCs with the G2/M phase marker Ser10 Phospho-Histone H3 (PH3) 37 revealed a significant reduction in the PH3-positive population in 5 μM PTC-209-treated SSCs, compared to the control group (Figure 2C and 2D). We next used TUNEL staining to determine the extent of apoptosis, and observed a dramatic increase in SSC apoptosis following treatment with 5 μM PTC-209 (Figure 2E and 2F). In accordance with the findings presented in Figure 1E-G, 1 μM PTC-209 had no obvious effects on the cell cycle (Figure 2A and 2B), cell proliferation (Figure 2C and 2D), or apoptosis (Figure 2E and 2F), although it did reduce the BMI1 knockdown efficiency in SSCs by 50% (Figure 1C and 1D).

To determine whether BMI1 is also involved in SSC differentiation, we next stained SSCs with two spermatogonia-specific differentiation markers, c-KIT and Stra8 38. We saw no obvious differences in the numbers of c-KIT- or Stra8-positive SSCs between the PTC-209-treated and negative control groups, suggesting that BMI1 was not required for SSC differentiation (Figure S1). Taken together, these results demonstrate that BMI1 likely controls cell cycle dynamics by disturbing cell proliferation and apoptosis, but does not affect the differentiation of SSCs.

### BMI1 binds near the transcriptional start site (TSS) of target genes

**Figure 3:**
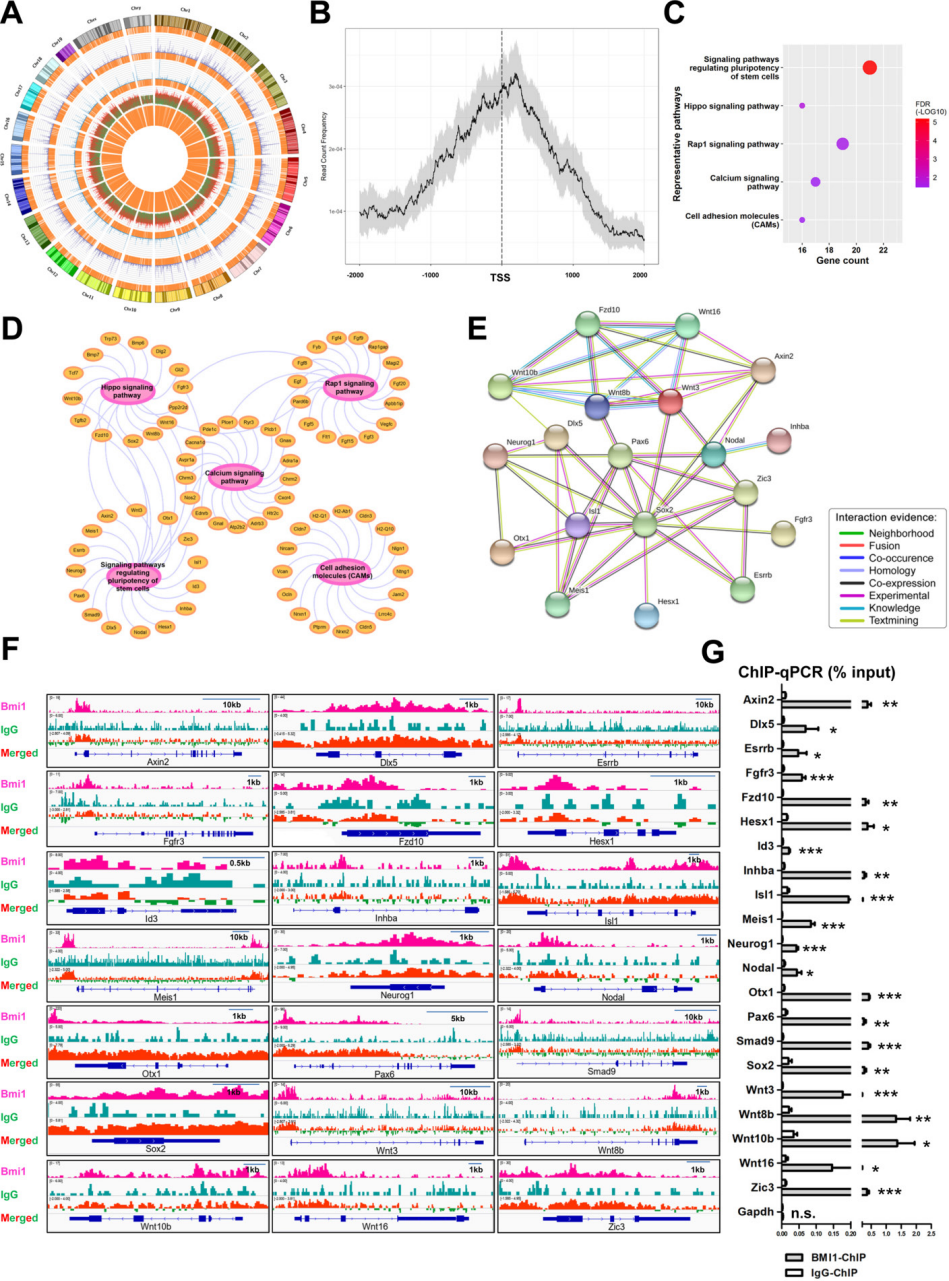
**BMI1-ChIP-Seq Analysis. (A)** Circos plot of public BMI1 ChIP-seq data. From the outside-in, the tracks represent chromosome karyotypes, peak distributions for the BMI1 group (purple), peak distributions for the IgG control group (blue), ratios of peak comparisons (the red or green color indicates higher or lower abundance in the BMI1 group) and the relative locations of annotated genes (orange). **(B)** Distribution of BMI1-binding sites relative to TSS of genes. **(C)** Representative KEGG pathway for BMI1's putative direct targets within 1 kb of their TSS. **(D)** Visualization of (C) based on Cytoscape Software.** (E)** Protein-protein association network involved in 'signaling pathways regulating pluripotency of stem cells' set of pathways was analyzed using the STRING database. **(F)** BMI1 binding peaks at the indicated gene loci.** (G)** ChIP-qPCR of BMI1 target genes in SSCs using an anti-BMI1 antibody. Data represent mean ± SD, n = 3 independently differentiated groups. Statistical analysis was performed using Student's two tailed t test. *P < 0.05; **P < 0.01; ***P < 0.001. not significant (n.s.), *P* > 0.05.

To explore the regulatory mechanism employed by BMI1 in SSC maintenance, we systemically re-analyzed the binding sites of BMI1 using public BMI1 ChIP-seq data generated using mouse germline stem cells (GSM1328932) 39 (Figure 3A). Interestingly, we observed that the majority of BMI1-binding sites were distributed within 1 kb of the TSS (Figure 3B), suggesting that BMI1 mainly bound to the promoter regions of target genes to regulate SSC maintenance. Kyoto Encyclopedia of Genes and Genomes (KEGG) pathway analysis for BMI1's putative direct targets (within 1 kb of their TSS) revealed a significant enrichment for genes involved in multiple signaling pathways, including signaling pathways regulating the pluripotency of stem cells, the Hippo, Rap1, Calcium signaling pathways, and Cell adhesion molecules (CAMs) (Figure 3C and 3D). As shown in Figure 3C, the “signaling pathways regulating the pluripotency of stem cells” was the most significantly enriched set of pathways, and was therefore selected for further analysis. The “signaling pathways regulating the pluripotency of stem cells” set of pathways contained 21 members, and their functional association networks were analyzed using the STRING database (https://string-db.org/) (Figure 3E); the BMI1-binding sites of the 21 genes are shown in Figure 3F. We next performed ChIP-qPCR analysis to validate the specific interactions of BMI1 and its direct targets using an anti-BMI1 antibody, and found that all of the 21 BMI1-binding peaks (Figure 3F) were precipitated by BMI1 in SSCs (Figure 3G). These findings provided us with clues for revealing the regulatory mechanisms employed by BMI1 in SSCs.

### BMI1 controls transcriptional repression through H2AK119ub and H3K4me3, independently of the PRC2 complex

**Figure 4:**
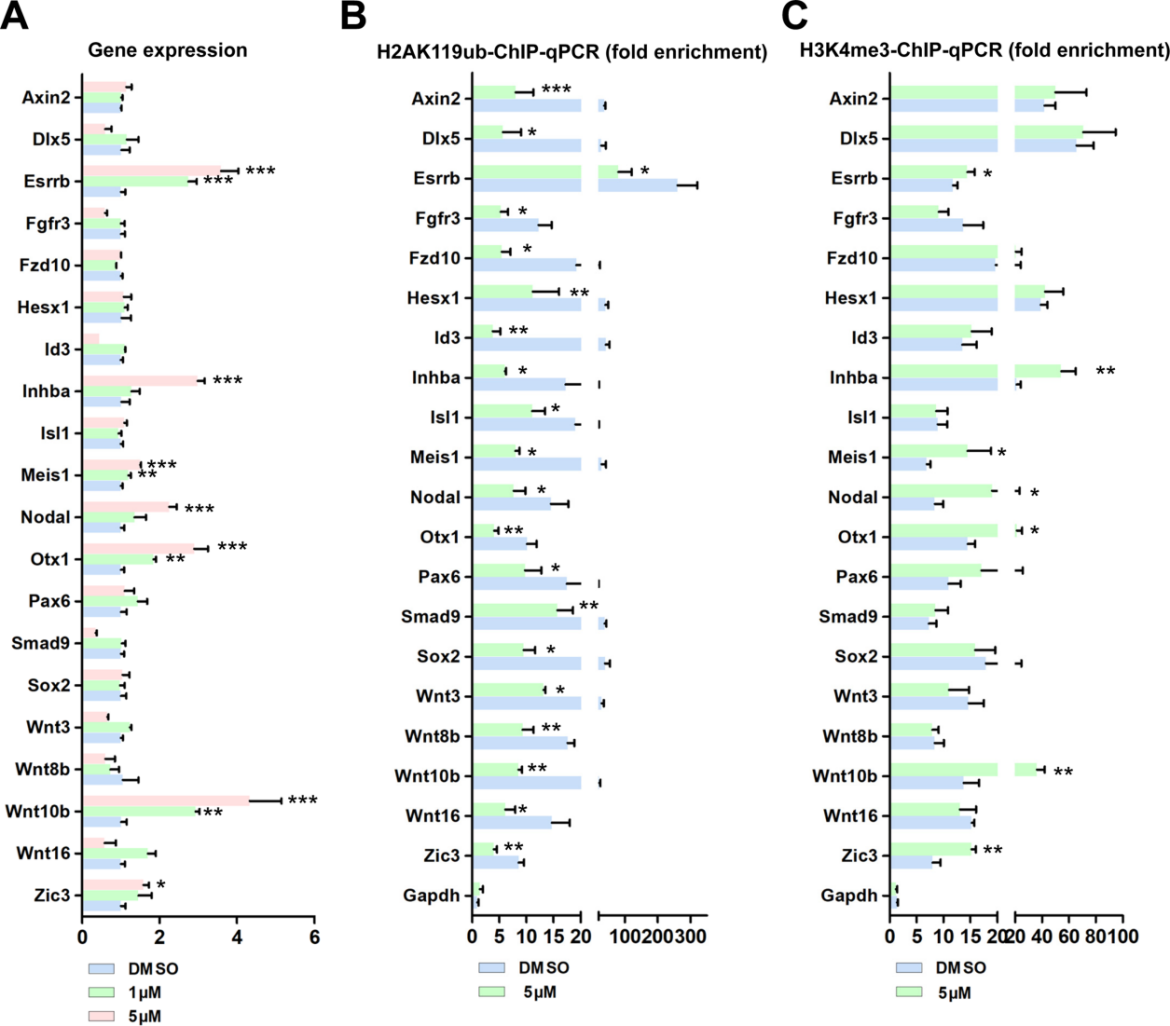
**BMI1 mediates transcriptional repression and modulates chromatin accessibility. (A)** Real-time PCR analysis of BMI1 targets in SSCs treated with DMSO, 1 µM, or 5 µM PTC-209 for 48 h. Among the 21 BMI1 target genes, the expressions of *Neurog1* in each group were undetectable and not proceeded for the further experiments. Data represent mean ± SD, n = 3 independently differentiated groups. Statistical analysis was performed using One-way ANOVA with Dunnett post hoc test. **(B-C)** ChIP-qPCR of BMI1 targets in SSCs treated with DMSO or 5 µM PTC-209 for 48 h using anti-H2AK119ub (B) and anti-H3K4me3 (C) antibodies. Data represent mean ± SD, n = 3 independently differentiated groups. Statistical analysis was performed using Student's two tailed t test. For (A) to (C), SSCs were separated from the feeder cells by gentle pipetting, and used for the indicated experiments. *P < 0.05; **P < 0.01; ***P < 0.001.

We next examined the effect of BMI1 on gene silencing, for the 21 BMI1 targets. Surprisingly, the PTC-209 treatment of SSCs only enhanced the mRNA expression of 7 (*Esrrb*, *Inhba*, *Meis1*, *Nodal*, *Otx1*, *Wnt10b*, and *Zic3*) out of the 21 BMI1 targets (Figure 4A). Of these targets, we observed that *Wnt10b* was dramatically up-regulated in a dose-dependent manner in PTC-209-treated SSCs (Figure 4A). Previous studies have provided evidence that the nuclear localized BMI1 monoubiquitinated histone H2A at K119 (H2AK119ub) and thereby mediated gene silencing 40, 41. Due to the nuclear localization of BMI1 in SSCs (Figure 1A), we therefore enquired whether BMI1 directly regulated transcriptional repression via H2AK119ub. To this end, we performed ChIP-qPCR with an anti-H2AK119ub antibody. As expected, after down-regulating BMI1 using PTC-209, H2AK119ub was eliminated from the binding peaks of all of the 21 BMI1 targets (Figure 4B), suggesting that the Polycomb protein function was disrupted upon BMI1 depletion.

However, these data still could not fully explain why only 7 out of the 21 BMI1 targets were transcriptionally repressed by BMI1 in SSCs. H3K4me3 marks the TSS of active genes 42. Previous studies from us and others have shown that H3K4me3 contributed to the derepression of BMI1-regulated genes, because reduced BMI1 expression corresponded with increased levels of the active histone mark H3K4me3 and reduced levels of repressive H2AK199ub at BMI1-binding loci 16, 43. We therefore performed ChIP-qPCR with an anti-H3K4me3 antibody, and found that H3K4me3 levels were only increased at the BMI1-binding sites of *Esrrb*, *Inhba*, *Meis1*, *Nodal*, *Otx1*, *Wnt10b*, and *Zic3* in PTC-209-treated SSCs (Figure 4C), suggesting that H3K4me3 contributes to the derepression of BIM1 targets.

Enhancer of zeste homolog 2 (EZH2) is the catalytic subunit of Polycomb repressive complex 2 (PRC2), which play conserved roles in catalyzing the transcriptional repression of target genes via the trimethylation of Lys27 on histone H3 (H3K27me3) 44, 45. We next sought to determine whether the PRC2 complex can partly compensate for the disrupted PRC1 complex in the regulation of transcriptional repression in PTC-209-treated SSCs. ChIP-qPCR demonstrated that there was no obvious compensation effect of EZH2 and H3K27me3 at the 21 BMI1 target loci in PTC-209-treated SSCs (Figure S2).

Taken together, these results indicate that BMI1 mediates transcriptional repression and modulates chromatin accessibility via H2AK119ub and H3K4me3, independently of the PRC2 complex.

### Wnt10b/ β-catenin signaling inhibits SSC maintenance

**Figure 5:**
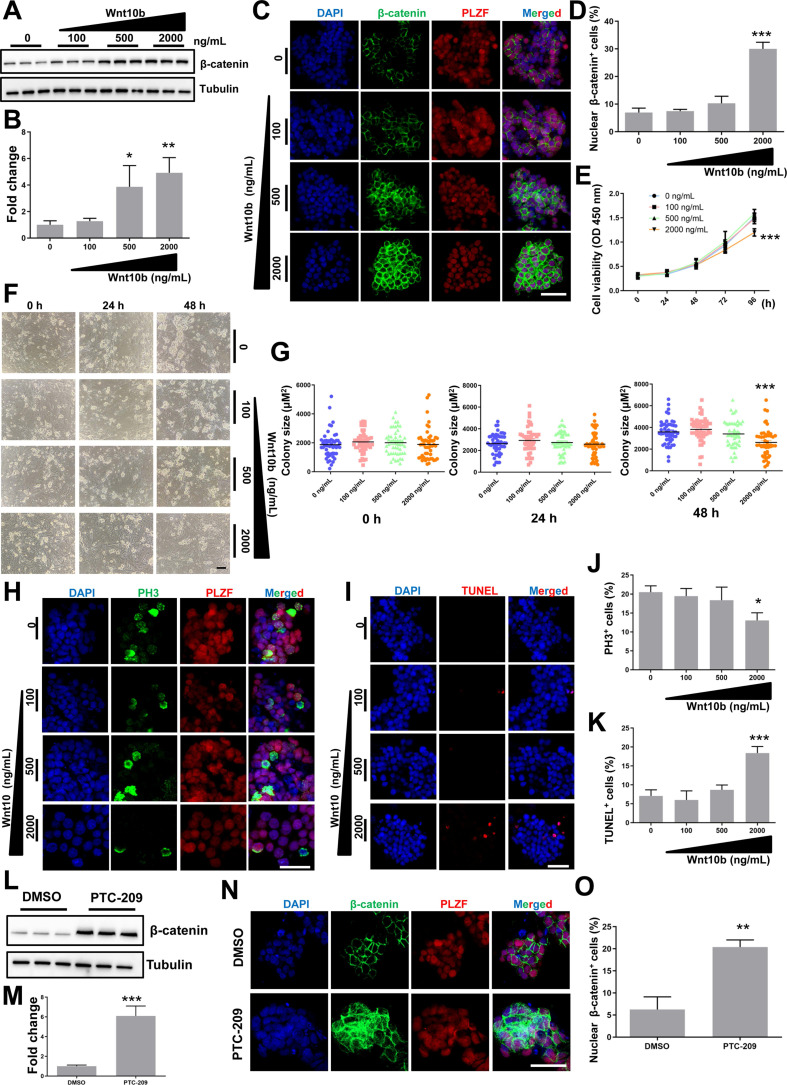
**The effect of Wnt10b on SSCs. (A)** SSCs were cultured in feeder-free conditions with the indicated doses of recombinant Wnt10b for 48 h. Cells were subsequently harvested and subjected for western blotting analysis.** (B)** Quantification of (A). Data represent mean ± SD, n = 3 independently differentiated groups. Statistical analysis was performed using One-way ANOVA with Dunnett post hoc test. **(C)** Co-immunostaining for β-catenin and PLZF in SSCs treated with the indicated doses of recombinant Wnt10b for 48 h, as described in (A). Scale bar, 50 µm.** (D)** Percentage of nuclear β-catenin-expressing cells in (C). Data represent mean ± SD, n = 3 independently differentiated groups. Statistical analysis was performed using One-way ANOVA with Dunnett post hoc test. **(E)** CCK-8 assay for SSCs treated with the indicated doses of recombinant Wnt10b under feeder-free conditions. The time point for Wnt10b treatment was identified as 0 h. Data represent mean ± SD, n = 3 independently differentiated groups. Statistical analysis was performed using One-way ANOVA with Dunnett post hoc test. **(F)** SSC colony analysis for SSCs treated with the indicated doses of recombinant Wnt10b for 48 h, as described in (A). The time point for the reintroduction of SSCs to feeder cells was identified as 0 h. Scale bar, 100 µm. **(G)** Quantification of SSC colony size of (F). Data represent mean ± SD, n = 50 colonies from three independently differentiated groups. Statistical analysis was performed using One-way ANOVA with Dunnett post hoc test. **(H)** Co-immunostaining for PH3 and PLZF in SSCs treated with the indicated doses of recombinant Wnt10b for 48 h, as described in (A). Scale bar, 50 µm. **(I)** TUNEL staining of SSCs treated with the indicated doses of recombinant Wnt10b for 48 h, as described in (A). Scale bar, 50 µm. **(J)** Quantification of (H). **(K)** Quantification of (I). For (J) and (K), data represent mean ± SD, n = 3 independently differentiated groups. Statistical analysis was performed using One-way ANOVA with Dunnett post hoc test. **(L)** SSCs were treated with DMSO or 5 µM PTC-209 for 48 h, and then removed from the feeder cells by gentle pipetting. Cells were lysed and subjected for western blotting analysis. **(M)** Quantification of (L). Data represent mean ± SD, n = 3 independently differentiated groups. Statistical analysis was performed using Student's two tailed t test. **(N)** Co-immunostaining for β-catenin and PLZF in SSCs treated with DMSO or 5 µM PTC-209 for 48 h.** (O)** Percentage of nuclear β-catenin-expressing cells in (N). Scale bar, 50 µm. Data represent mean ± SD, n = 3 independently differentiated groups. Statistical analysis was performed using Student's two tailed t test. For (B), (D), (E), (G), (J), (K), (M), and (O), *P < 0.05; **P < 0.01; ***P < 0.001.

Because *Wnt10b* was the most up-regulated target of BMI1 in PTC-209-treated SSCs (Figure 4A), we further investigated the role of *Wnt10b* in SSCs. To circumvent the indirect action of the Wnt10b recombinant protein on feeder cells, SSCs were separated from feeder cells by gentle pipetting and then cultured in feeder-free conditions with or without the Wnt10b recombinant protein. We observed that recombinant Wnt10b treatment enhanced β-catenin expression in a dose-dependent manner (Figure 5A and 5B), which led to the nuclear translocation of β-catenin in SSCs (Figure 5C and 5D), indicating active Wnt/β-catenin signaling. Moreover, SSC growth was significantly inhibited by the administration of recombinant Wnt10b (at 2,000 ng/mL) for 96 hours (Figure 5E). To evaluate the effect of recombinant Wnt10b on colony formation, feeder-free SSCs were treated with recombinant Wnt10b for 48 hours and subsequently co-cultured with feeder cells, because feeder cells promote colony formation. SSC colony size was reduced following the addition of recombinant Wnt10b, even after reintroduction of feeder cells (Figure 5F and 5G). Meanwhile, we used PH3 and TUNEL staining to evaluate the extent of cell proliferation and apoptosis, respectively, and found a large reduction in PH3-positive and a dramatic increase in TUNEL-positive SSC populations in the Wnt10b (2000 ng/mL) treatment group (Figure 5H-5K), which closely mimicked the phenotype of PTC-209-treated SSCs (Figure 1E-1G, and Figure 2C-2F). In order to confirm that 2000 ng/mL Wnt10b was sufficient to induce SSCs apoptosis as found in Figure 5I, we also used Annexin-V/PI staining to examine SSCs apoptosis (Figure S3). And the results were highly consistent with the TUNEL assay (Figure 5I).

To assess the relationship between BMI1 and Wnt/β-catenin signaling, we further examined changes in β-catenin levels after reducing BMI1 expression, and found that β-catenin was markedly up-regulated in PTC-209-treated SSCs (Figure 5L and 5M). As expected, β-catenin was ectopically activated and the numbers of nuclear β-catenin-positive cells were significantly increased after treatment with PTC-209 (Figure 5N and 5O), indicating that BMI1 inhibition could also directly activate Wnt/β-catenin signaling in SSCs. Taken together, we conjectured that BMI1 directed SSC maintenance via the repression of Wnt10b/β-catenin signaling.

### XAV939 restores β-catenin expression and SSC maintenance in PTC-209-treated SSCs

**Figure 6:**
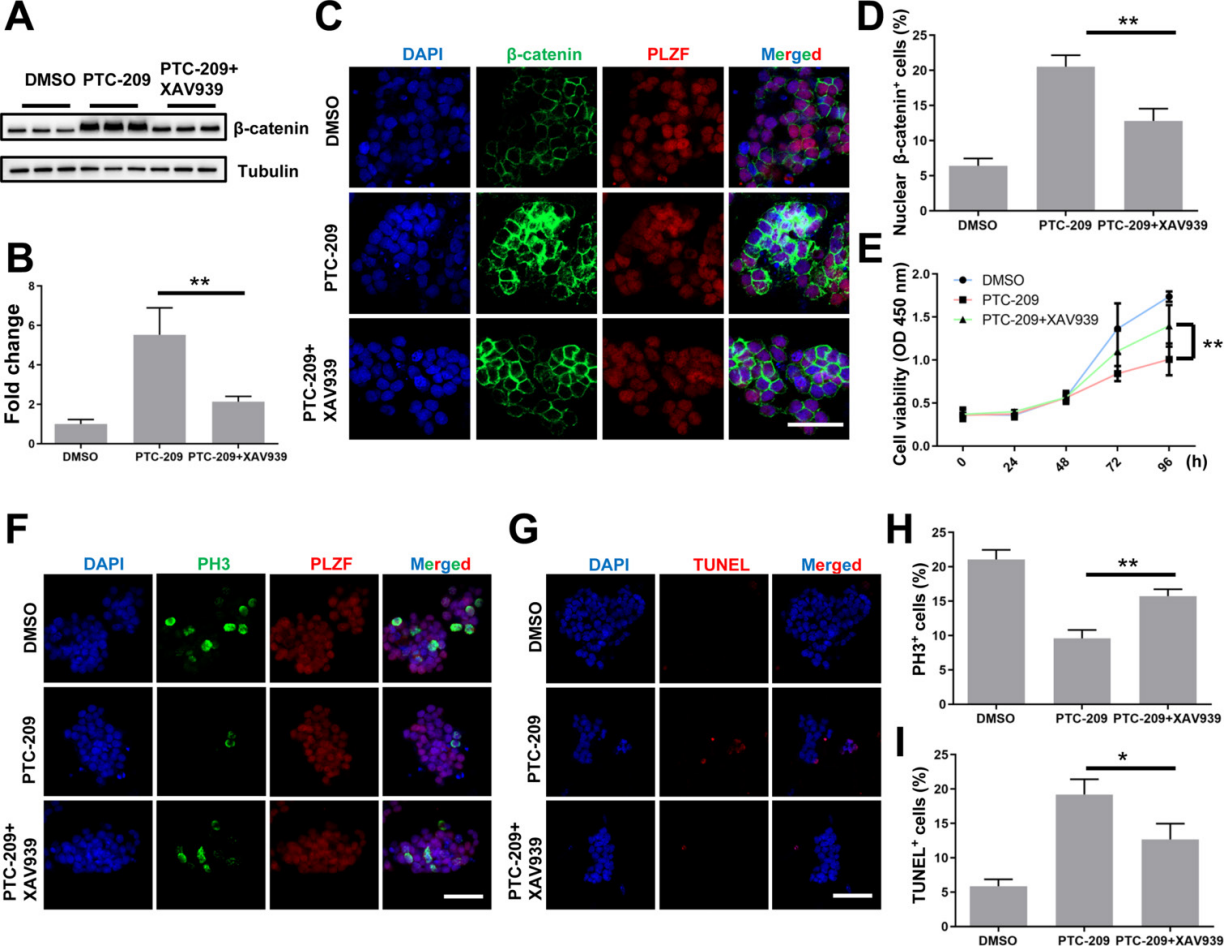
**XAV939 rescues the altered phenotypes of PTC-209-treated SSCs. (A)** SSCs were treated with DMSO or PTC-209 for 48 h, separated from the feeder cells and subsequently cultured under feeder-free conditions with or without XAV939 for 48 h. Cells were lysed and subjected to western blotting analysis. **(B)** Quantification of (A). Data represent mean ± SD, n = 3 independently differentiated groups. **(C)** Co-immunostaining for β-catenin and PLZF of SSCs treated with DMSO, PTC-209, or PTC-209 + XAV939, as described in (A). Scale bar, 50 µm. **(D)** Percentage of nuclear β-catenin-expressing cells in (C). Data represent mean ± SD, n = 3 independently differentiated groups.** (E)** CCK-8 analysis of SSCs treated with DMSO, PTC-209, or PTC-209 + XAV939, at the indicated time points. The time point for XAV939 treatment was identified as 0 h. Data represent mean ± SD, n = 6 independently differentiated groups. **(F)** Co-immunostaining for PH3 and PLZF in SSCs treated with DMSO, PTC-209, or PTC-209 + XAV939, as described in (A). Scale bar, 50 µm. **(G)** TUNEL staining of SSCs treated with DMSO, PTC-209, or PTC-209 + XAV939, as described in (A). Scale bar, 50 µm. **(H)** Quantification of (F). Data represent mean ± SD, n = 3 independently differentiated groups. **(I)** Quantification of (G). Data represent mean ± SD, n = 3 independently differentiated groups. For (A) to (I), PTC-209 and XAV939 were used at the concentrations of 5 µM and 20 µM, respectively. Statistical analysis was performed using One-way ANOVA with Dunnett post hoc test. *P < 0.05; **P < 0.01.

To confirm whether aberrant Wnt/β-catenin pathway activation contributed to the SSC maintenance defects observed in PTC-209-treated cells, we used a specific Wnt/β-catenin pathway inhibitor, XAV939 32, 46, to promote the degradation of β-catenin in PTC-209-treated SSCs. To determine the inhibitory efficiency of XAV939, SSCs were treated with three concentrations (5, 10, and 20 μM) of XAV939. Western blotting analysis showed that XAV939 could suppress β-catenin level in a dose-dependent manner (Figure S4A and S4B). As expected, β-catenin expression and distribution could be recovered following the administration of XAV939 (20 μM) to PTC-209-treated SSCs, when compared to PTC-209 treatment alone (Figure 6A-6D). Moreover, XAV939 treatment could also restore the growth (Figure 6E), proliferation, and apoptosis (Figure 6F-6I) of PTC-209-treated SSCs, in contrast to PTC-209 treatment alone, while XAV939 treatment alone had no obvious effect on SSC viability (Figure S4C). The above data imply that the elimination of over-activated β-catenin signaling in PTC-209-treated SSCs is a very effective approach for reversing SSC maintenance defects.

### BMI1 deficiency causes SSC maintenance defects in murine testes

**Figure 7:**
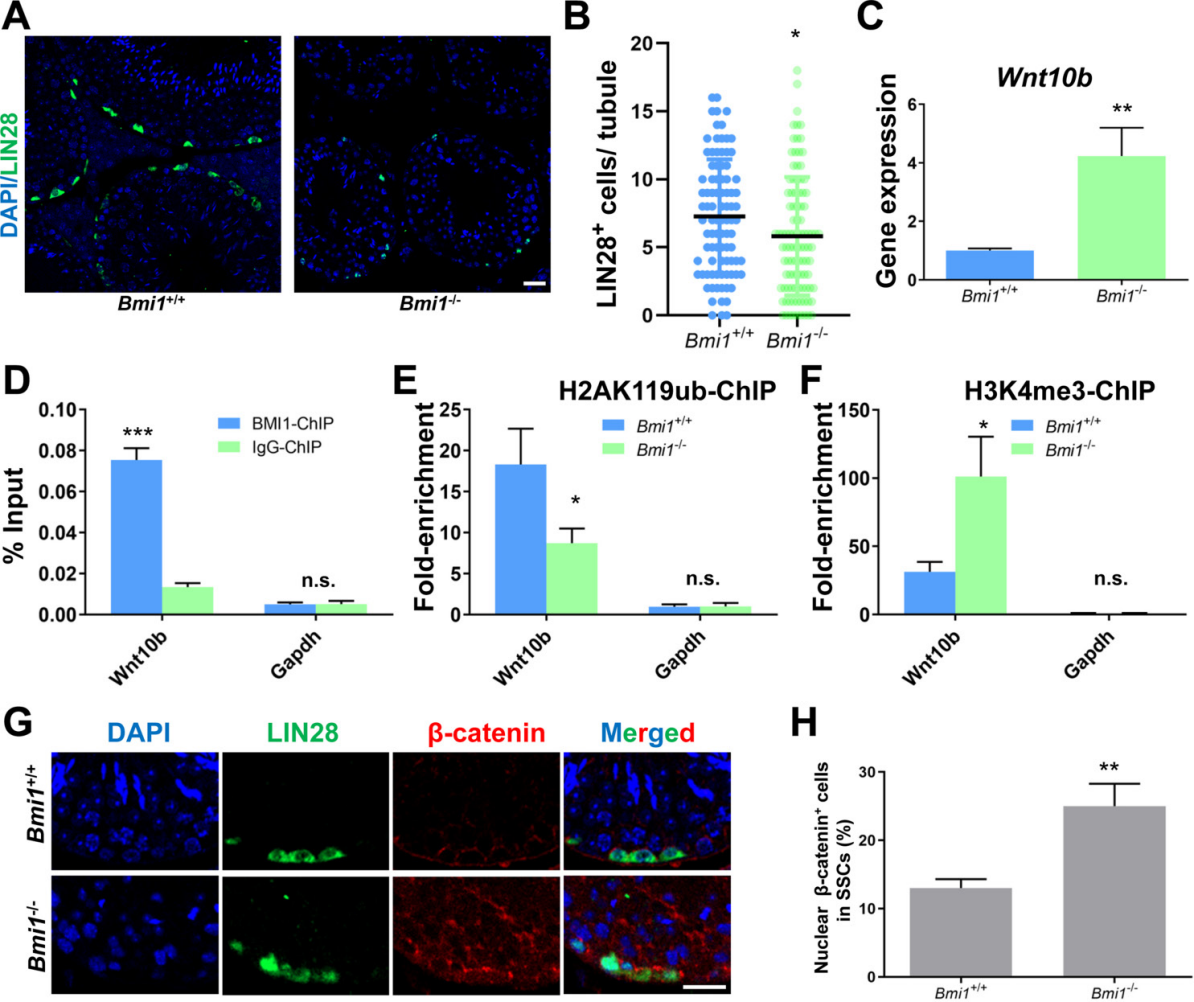
** BMI1 epigenetically regulates SSC maintenance via Wnt10b and β-catenin in murine testes. (A)** Immunostaining for LIN28 in *Bmi1^+/+^* and *Bmi1^-/-^* murine testes. Scale bar, 20 µm. **(B)** Quantification of (A). Data represent mean ± SD, n = 91 tubules from three mice from *Bmi1^+/+^* group and n = 100 tubules from three mice for *Bmi1^-/-^* group. **(C)** Real-time PCR analysis of *Wnt10b* in *Bmi1^+/+^* and *Bmi1^-/-^* mouse testes. Data represent mean ± SD, n = 3 independently differentiated groups. **(D)** ChIP-qPCR of *Wnt10b* in murine testes using an anti-BMI1 antibody. Data represent mean ± SD, n = 3 independently differentiated groups. **(E-F)** ChIP-qPCR of *Wnt10b* in *Bmi1^+/+^* and* Bmi1^-/-^* mouse testes using anti-H2AK119ub (E) and anti-H3K4me3 (F) antibodies. Data represent mean ± SD, n = 3 independently differentiated groups.** (G)** Co-immunostaining for LIN28 and β-catenin in *Bmi1^+/+^* and* Bmi1^-/-^* murine testes. Scale bar, 20 µm. **(H)** Percentage of nuclear β-catenin-expressing cells in LIN28-labeled SSCs. Data represent mean ± SD, n = 3 independently differentiated groups. For (B), (C), (D), (E), (F), and (H), statistical analysis was performed using Student's two tailed t test. *P < 0.05; **P < 0.01. not significant (n.s.), *P* > 0.05.

**Figure 8:**
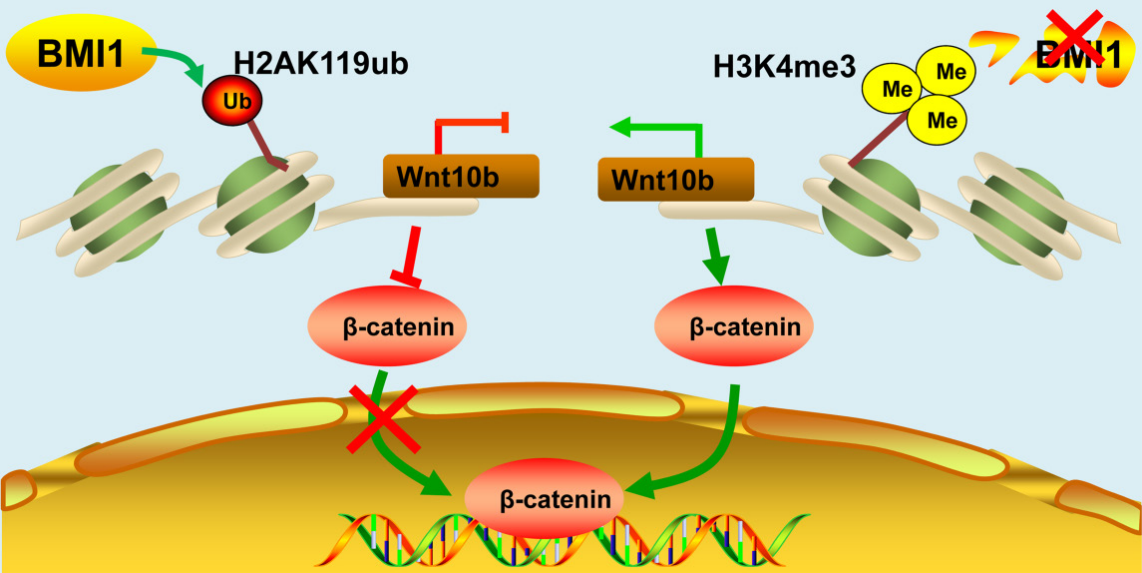
**Schematic illustration of BMI1 in SSCs.** BMI1 is required for SSC maintenance by facilitating the monoubiquitination of H2AK119 to repress *Wnt10b* expression*,* and thereby inactivates the Wnt/β-catenin signaling*.* In the absence of BMI1, *Wnt10b* is derepressed via H3K4me3, which promotes the nuclear translocation of β-catenin.

We next conducted experiments in the testes of *Bmi1* knock-out mice to further support our *in vitro* findings. We observed that the number of SSCs labeled with LIN28 47 was significantly lower in the testes of *Bmi1* knockout (*Bmi1^-/-^*) compared to wild-type (*Bmi1^+/+^*) mice (Figure 7A and 7B), which might be a result of compromised proliferation and excessive apoptosis in *Bmi1^-/-^* mice (Figure S5), indicating the requirement for BMI1 in SSC maintenance. qRT-PCR showed that the relative *Wnt10b* mRNA expression level was up-regulated in* Bmi1^-/-^* murine testes (Figure 7C). As expected, the BMI1-binding site of *Wnt10b* could be precipitated using an anti-BMI1 antibody (Figure 7D). Meanwhile, H2AK119ub was disaggregated and H3K4me3 levels were dramatically increased at the BMI1-binding sites of *Wnt10b* in the testes of *Bmi1^-/-^* mice (Figure 7E and 7F). Furthermore, by co-staining with LIN28 and β-catenin, we found that nuclear β-catenin-positive SSC numbers significantly increased in the testes of *Bmi1* knock-out animals (Figure 7G-H). Combined with our *in vitro* findings, we demonstrated that BMI1 is required for SSC maintenance by epigenetically repressing *Wnt10b,* and mediating Wnt/β-catenin signaling (Figure 8).

## Discussion

SSCs balance the self-renewal and differentiation processes that maintain the stem cell pool and sustain continuous sperm production 48. However, the regulatory mechanisms involved in the maintenance of SSCs are poorly understood. In this study, we uncover a novel regulatory mechanism for SSC maintenance, involving the protein BMI1, and reveal that BMI1 modulates chromatin accessibility via Wnt10b-mediated β-catenin signaling.

BMI1 can be activated either in the cytoplasm or in the nucleus via multiple regulatory mechanisms. It has been shown that BMI1 regulates stem cell function by maintaining mitochondrial activity and redox homeostasis 13. Another study has shown that BMI1 could localize to the inner mitochondrial membrane and directly regulate mitochondrial function by stabilizing mtRNA homeostasis and bioenergetics 49. Nevertheless, in this study, we found that BMI1 resided in the nuclei of SSCs, indicating the epigenetic inhibitory function of this protein in SSC maintenance. Importantly, BMI1-mediated epigenetic silencing in SSCs not only resulted in a lower distribution of H2AK119ub but also led to an increase in the distribution of H3K4me3 at several key BMI1 targets. Interestingly, our data also indicate that H3K4me3 was required for the derepression of BIM1 targets, which was consistent with our previous findings in testicular Leydig cells 16.

Recent studies have indicated that the action of Wnt/β-catenin signaling was more complex than previously thought, and that β-catenin was able to induce various reproductive phenotypes in different mouse models. For instance, the genetic elimination of *β-catenin* in undifferentiated spermatogonia under the control of Axin2 (CreERT2) resulted in testicular atrophy and decreased the proliferation of undifferentiated spermatogonia, subsequently triggering germ cell maintenance defects 28. In addition, the spermatid-specific deletion of *β-catenin* under the control of Prm1-Cre led to significant germ cell loss, increased germ cell apoptosis, and impaired fertility 50. In contrast, the deletion of *β-catenin* in differentiating spermatogonia and early spermatocytes under the control of Stra8-Cre did not affect male fertility and spermatogenesis 51. Apart from the loss-of-function studies of *β-catenin* in germ cell development, gain-of-function studies of *β-catenin in vivo* also elicit conflicting data. It was reported that the constitutive activation of β-catenin in the primordial germ cells (PGCs) in the Ctnnb1^tm1Mmt/tm1Mmt^; TNAP^cre/+^ model markedly reduced PGC numbers and induced cycle arrest in these cells 52. A subsequent study using a Ctnnb1^tm1Mmt/tm1Mmt^; Ddx4^cre/+^ model showed that the constitutive activation of β-catenin in fetal gonocytes triggered SSC proliferation and spermatocyte depletion 53. However, using the Ctnnb1^tm1Mmt/tm1Mmt^; Ddx4^cre/+^ model, Boyer *et al*., found that the constitutive activation of β-catenin in fetal gonocytes compromised SSCs activity and promoted SSCs differentiation 54. In addition, the constitutive activation of β-catenin in fetal gonocytes using a Ctnnb1^tm1Mmt/tm1Mmt^; Nanos3^cre/+^ model reduced the GFRα1^+^ SSC pool 55. In addition to its direct role in germ development, β-catenin signaling also plays crucial roles in soma-germline communications in the male stem cell niche. For example, the stabilization of β-catenin in fetal Sertoli cells using the Ctnnb1^tm1Mmt/tm1Mmt^; Amh^cre/+^ mouse model led to testis malformation and germ cell depletion from embryonic day 15.5 onwards 56. Similarly, constitutive β-catenin signaling in neonatal Sertoli cells, studied using the Ctnnb1^tm1Mmt/tm1Mmt^; Amhr2^cre/+^ mouse model caused a progressive loss of germ cells and attenuated SSC activity due to SSC niche disruption 57-59. Nevertheless, another group reported that Sertoli cells uniquely secreted Wnt6 to direct β-catenin signaling activation and subsequently promote the proliferation of SSCs in the murine testes 28. Taken together, the different observations from the aforementioned studies suggest that the precise regulation of Wnt/β-catenin signaling in spermatogenesis is still not well understood. Of note, the above studies predominantly focused on the effects of β-catenin, while largely sidelining the upstream regulatory mechanisms implicated.

In this study, we systematically explored the role of the BMI1/Wnt/β-catenin signaling axis in SSC maintenance, using both *in vitro* and *in vivo* approaches. Unlike the pro-proliferative activity of Wnt6 in SSCs 28, we found that Wnt10b exerted an anti-proliferative effect, revealing a novel regulatory mechanism directed by BMI1/Wnt10b/β-catenin signaling in SSCs. Wnt10b has been shown to have roles in the maintenance of normal mitotic microtubule dynamics and appropriate chromosome segregation via the Wnt10b/GSK3β-driven cellular machinery 60. However, little is known about Wnt10b's role in regulating SSC characteristics. Here, we discovered that recombinant Wnt10b could dose-dependently up-regulate β-catenin and enhance the nuclear translocation of β-catenin in SSCs, thereby aggravating SSC maintenance defects. To support this hypothesis, we also showed that the acceleration of β-catenin degradation by XAV939 was sufficient to reverse the altered phenotypes of PTC-209-treated SSCs. Thus, our study complements the available data relating to the Wnt/β-catenin-associated regulatory mechanism in SSCs.

In conclusion, to the best of our knowledge, the present study for the first time reveals the major role of BMI1 in SSC maintenance via autonomous cell effects. Using *in vitro* and *in vivo* approaches, we revealed a novel regulatory mechanism of BMI1-mediated Wnt/β-catenin signaling in SSCs, providing potential therapeutic targets for the clinical diagnosis and treatment of SSC maintenance defects.

## Supplementary Material

Supplementary figures and tables.
