# Supplementary material for: BMI1 promotes spermatogonial stem cell maintenance by epigenetically repressing Wnt10b/β-catenin signaling
Source: Int J Biol Sci. 2022 Apr 4;18(7):2807–20. doi: 10.7150/ijbs.70441 (PMC9066105; doi:10.7150/ijbs.70441)
Supplement: Supplementary file 1 — Supplementary figures and tables. [file ijbsv18p2807s1.pdf]

Supplementary Material

Supplementary Figures and Tables

Table S1. Antibodies used in the study

Antigen	Source	Company	Application	Dilution
BMI1	Rabbit	Proteintech	WB; IF; ChIP	1:1000; 1:200; 10μg/ChIP
PLZF	Goat	R&D	IF	1:1000
Tubulin	Mouse	Beyotime	WB	1:10000
ub-Histone H2A(K119)	Rabbit	Cell Signal	ChIP	10μg/ChIP
PH3	Rabbit	Cell Signal	IF	1:1000
β-catenin	Mouse	BD	WB;IF	1:1000;1:500
LIN28	Rabbit	Abcam	IF	1:500
Stra8	Rabbit	Millipore	IF	1:500
EZH2	Rabbit	Cell Signal	ChIP	5μg/ChIP
H3K4me3	Rabbit	Millipore	ChIP	5μg/ChIP
c-KIT	Goat	R&D	IF	1:500
H3K27me3	Rabbit	Millipore	ChIP	5μg/ChIP

Table S2: The list of primer sequences for gene expression analysis.

Gene	Forward (5'->3')	Reverse (5'->3')	Product length (bp)
Axin2	GGTCCTGGCAACTCAGTAACA	CTCATGTGAGCCTCCTCTCTTTT	144
Dlx5	CCGTCTCAGGAATCGCCAAC	CTCCGCCACTTCTTTCTCTGG	285
Esrrb	TTGCATGGGGCTCTGTG	GGTTCAGCAGCATGGTTGG	300
Fgfr3	GCGACAGGTGTCCTTGGAA	CTTGGCAGTACGGTCCTTGT	244
Fzd10	TGTGACAACCCAGGCAAGTT	CGATGGGTCGATGAGGAAGG	189
Hesx1	TTTCACCCAGAACCAGGTCG	TTGCTCGGCGATTTTGGAAC	143
Id3	CATCTCCCGATCCAGACAGC	GTTCAGTCCTTCTCTCGGGC	266
Inhba	GGTGCTGCTCAAGTGCCAATA	CTGGCAGCAAAAGTCGTGTG	208
Isl1	TCTGCAAATGGCAGCCGAA	GTCCTTGCACCGCTTGTTTT	244
Meis1	ACTGCTGTCTTGGTGGAACC	TAGGTCGTCGTACCTTTGCG	266
Nodal	AGGGACCCTGGTGTTGTTTG	CCGGTCACGTCCACATCTTG	251
Otx1	GGGAGGGCAGTAGAAAGGTG	GCCTGGCCTGCCCTC	162
Pax6	CACCAGACTCACCTGACACC	TCACTCCGCTGTGACTGTTC	193
Smad9	CACCTCTCGGACAGAGTAAGC	GCAAGTACCTGTCCACGGAT	141
Sox2	TTTGTCCGAGACCGAGAAGC	ATCATGCTGTAGCTGCCGTT	279
Wnt3	TGTTCCAACTATTGGGGGCG	GGCCAGGGACCACCAAAT	163
Wnt8b	TGACCGGTCCAAAGGCTTAC	CCCAGTTGTCCATTTCGGGA	280
Wnt10b	GAGTTAGGTCGAGCAGAGCC	GTCAGTCAGCGCCTCCAG	286
Wnt16	GCAGGCAACATGACCGAATG	CATTAACTTGGCGACAGCCTGC	231
Zic3	CGCGCTCTTGAGTAGAGGAG	CCGGAGCTGCAACTTTCCTA	248
Neurog1	GCTGCCCTCGGTCTATTTCT	GCCGGCTCAAACCGAATTTT	261
18s rRNA	AAACGGCTACCACATCCAAG	CCTCCAATGGATCCTCGTTA	155

Table S3: The list of primer sequences for ChIP-qPCR.

Gene	Forward (5'->3')	Reverse (5'->3')	Product length (bp)
Axin2	CAGCCAAGCTCTCCGGTTTA	GGAACCTGGGTCTTCATGGG	115
Dlx5	AGCCCTAGTGGTGTTTGCGT	GCGCTTCGCTGGCTAATCC	99
Esrrb	CTCCTACAGACTCCTCCCCC	CAAGTACTGTGGGAGCTGGG	116
Fgfr3	TGCCCATACATGACACGTCC	CTTCCAACTCCACAGAGCCC	78
Fzd10	CTTCCTCATCGACCCATCGC	ATAGCCCACCGAATAAACGCA	88
Hesx1	GGAAGCAGACTCCGCTAGG	AGACAAGAGAGAGAACAGAGTTGC	70
Id3	TGCTGCCTGTCGGAACG	GGCTAAGAGGCTCCTCGGT	76
Inhba	GGATGCACTTTGGATATTCAGGAC	CCAGTCGATATTCACCTGTGCT	80
Isl1	TCCGGGTTTGTCCCCTAAC	CTTCACTGACTCCCGTGCT	116
Meis1	CGTGCGAATTTGTGGAGTGT	ACAGGTCGCGTCTTACACAAT	121
Nodal	CCCTCTGGCGTACATGTTGA	AGGCTTGCAGCCTACCTTG	92
Otx1	GCTCCTTAGGGCAAGCAAGA	AGGAGAAAGTTCTGCACCCG	151
Pax6	AGGAAAGTTAGCGCCTGCC	GGTTCTACGCGAGGACCTG	80
Smad9	TACCATTGGGTGCCAGCG	AGAAGTGCAGCGATTAGCCAC	130
Sox2	TAAGTACACGCTTCCCGGAG	ATCATGCTGTAGCTGCCGTT	153
Wnt3	TCCCGGAGAAAAGCGAGGT	CCGTCTTCCAAAAGCAACGAA	123
Wnt8b	TCGTGAATGAGCCTAGCGAT	AATCGGGAATGTTGCAACTCG	104
Wnt10b	AGTGGGGCTAATCGTGATCC	GACTTGACCAGGAGAACGCAG	93
Wnt16	GCGCGTTCCTTCGATAGAGT	CACCTCGCACCAAATGTTCC	83
Zic3	ACCGCAGGGTTCAGGTTATG	GCGCGTGGAGTTGAAAGC	121
Neurog1	CTCCAAACCTCCTGTCCGTC	GCTGGAGCAGTCGAGATCAG	90
Gapdh	AACCCAAACTAACAGTTGTCCCAA	ACTCCTTGGAGGCCATGTAGG	102

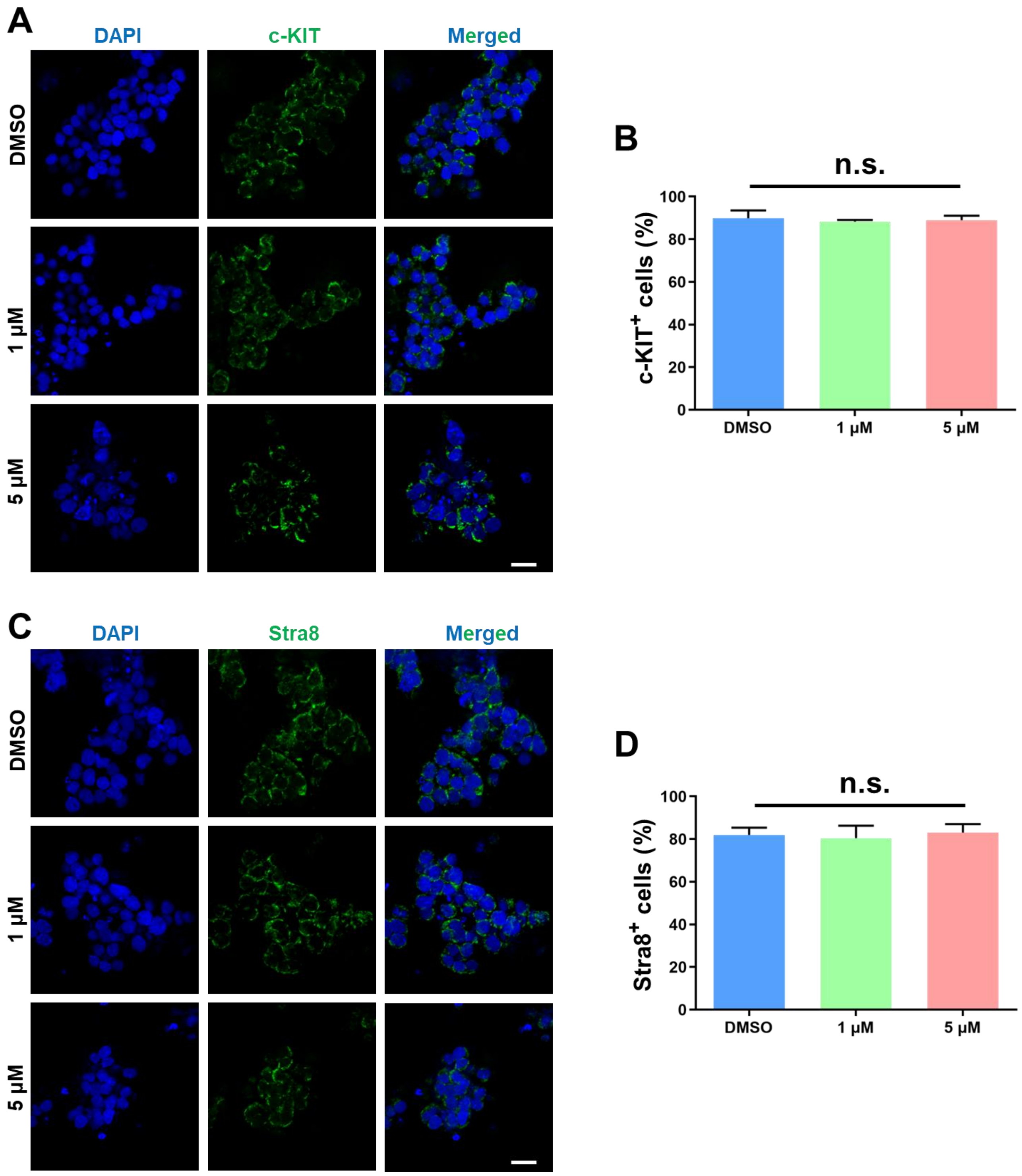

Figure S1. PTC-209 has no effect on SSC differentiation upon retinoic acid treatment. (A) Immunostaining for c-KIT in SSCs treated with DMSO, 1 μM, or 5 μM PTC-209 for 48 h. **(B)** Quantification of (A). **(C)** Immunostaining for Stra8 in SSCs treated with DMSO, 1 μM, or 5 μM

PTC-209 for 48 h. **(D)** Quantification of (C). For (A) to (D), SSCs were treated with 1 μM retinoic acid for 48h.Data represent mean ± SD, n = 3 independently differentiated groups. Statistical analysis was performed using One-way ANOVA with Dunnett post hoc test. not significant (n.s.), *P* > 0.05. Scale bar, 20 μm.

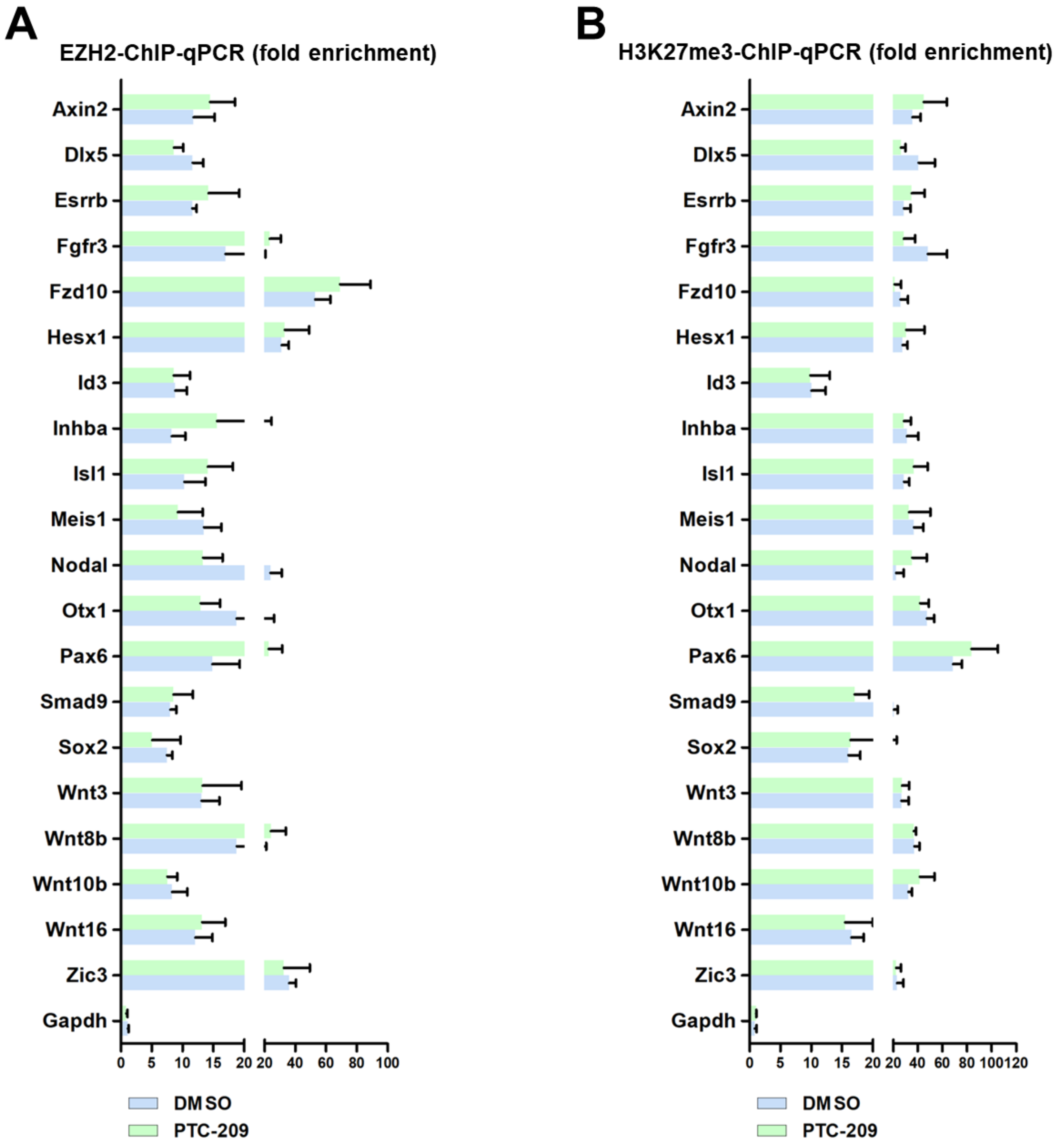

Figure S2. Distribution of EZH2 and H3K27me3 at the BMI1 targets. (A-B) ChIP-qPCR of BMI1 target genes in SSCs treated with DMSO or 5 μM PTC-209 for 48 h using anti-EZH2 (A) and anti-H3K27me3 (B) antibodies. Data represent mean ± SD, n = 3 independently differentiated groups. Statistical analysis was performed using Student's two tailed t test. For all tests, $P > 0.05$.

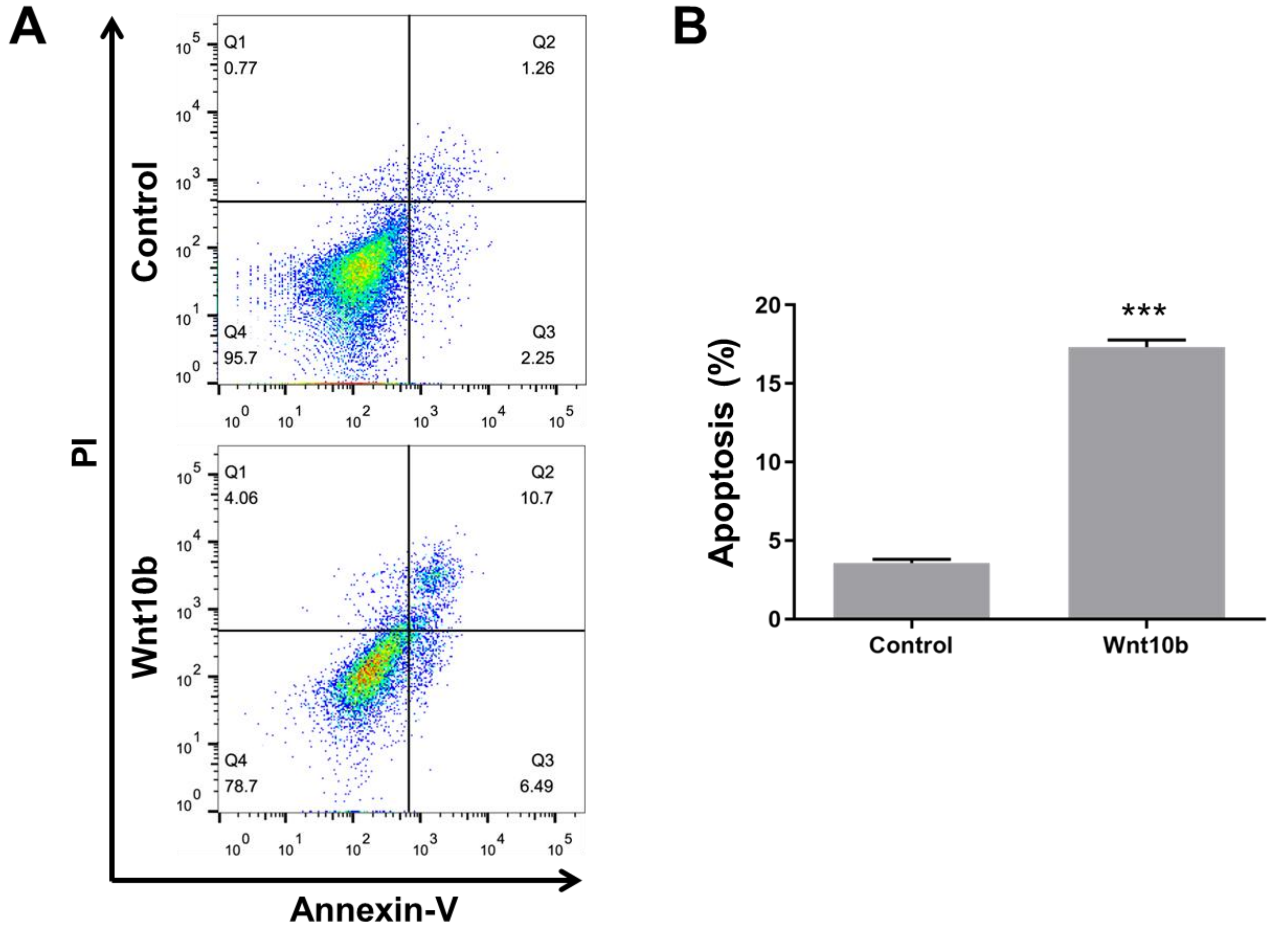

Figure S3. The effect of Wnt10b on SSC apoptosis. (A) Flow cytometry-based Annexin V-FITC/PI staining of SSCs treated with PBS (control) or 2000 ng/mL Wnt10b for 48 h. **(B)** Quantification of Q2 + Q3 in (A). Data represent mean ± SD, n = 3 independently differentiated groups. Statistical analysis was performed using Student's two tailed t test. ****P* < 0.001.

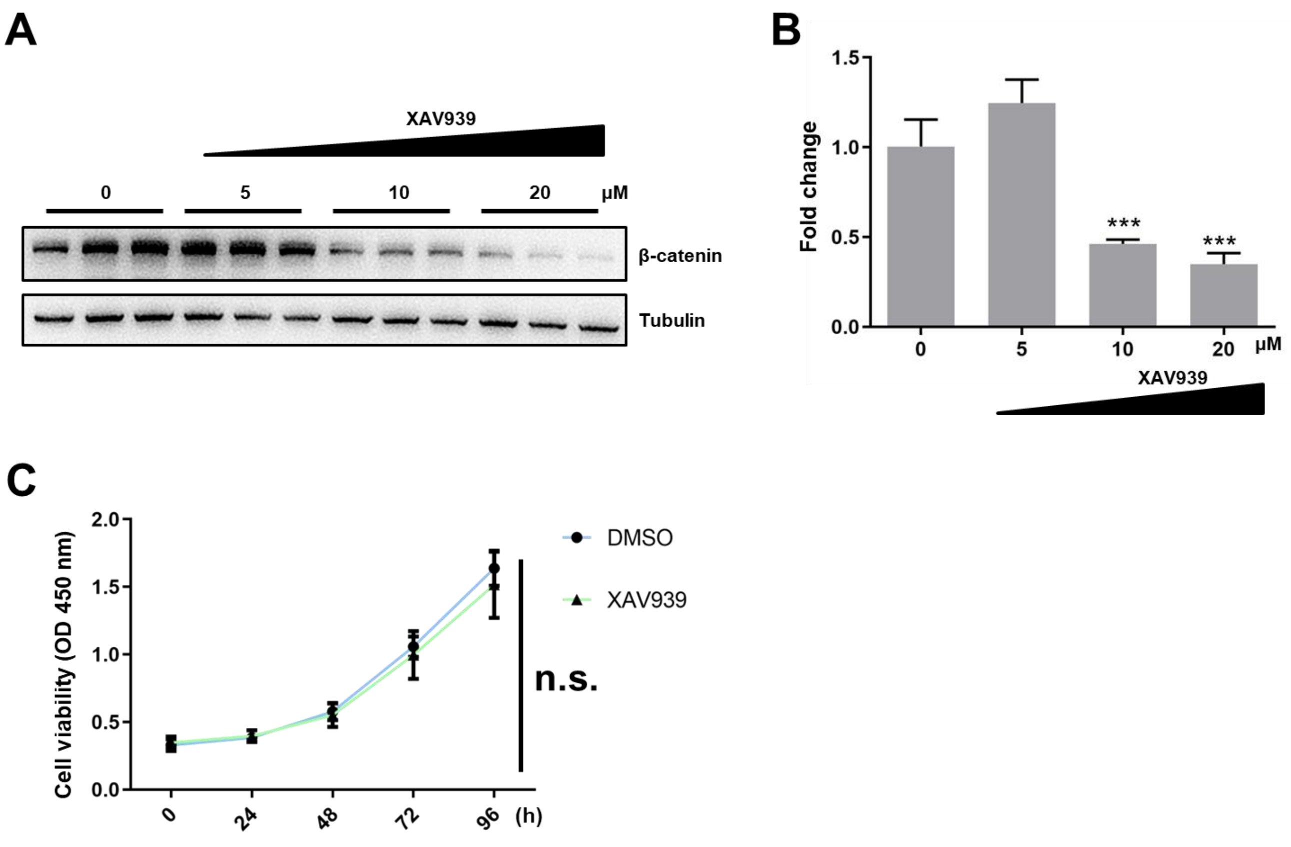

Figure S4. XAV939 treatment alone has no effect on SSC viability. (A) SSCs were cultured in feeder-free conditions with the indicated doses of XAV939 for 48 h. Cells were subsequently harvested and subjected for western blotting analysis. **(B)** Quantification of (A). Data are presented as mean ± SD, n = 3 independently differentiated groups. Statistical analysis was performed using One-way ANOVA with Dunnett post hoc test. **(C)** CCK-8 assay for SSCs treated with DMSO or 20 μM XAV939 under feeder-free conditions. The time point for XAV939 treatment was identified as 0 h. Data represent mean ± SD, n = 6 independently differentiated groups. Statistical analysis was performed using Student's two tailed t test. ***$P < 0.001$. not significant (n.s.), $P > 0.05$.

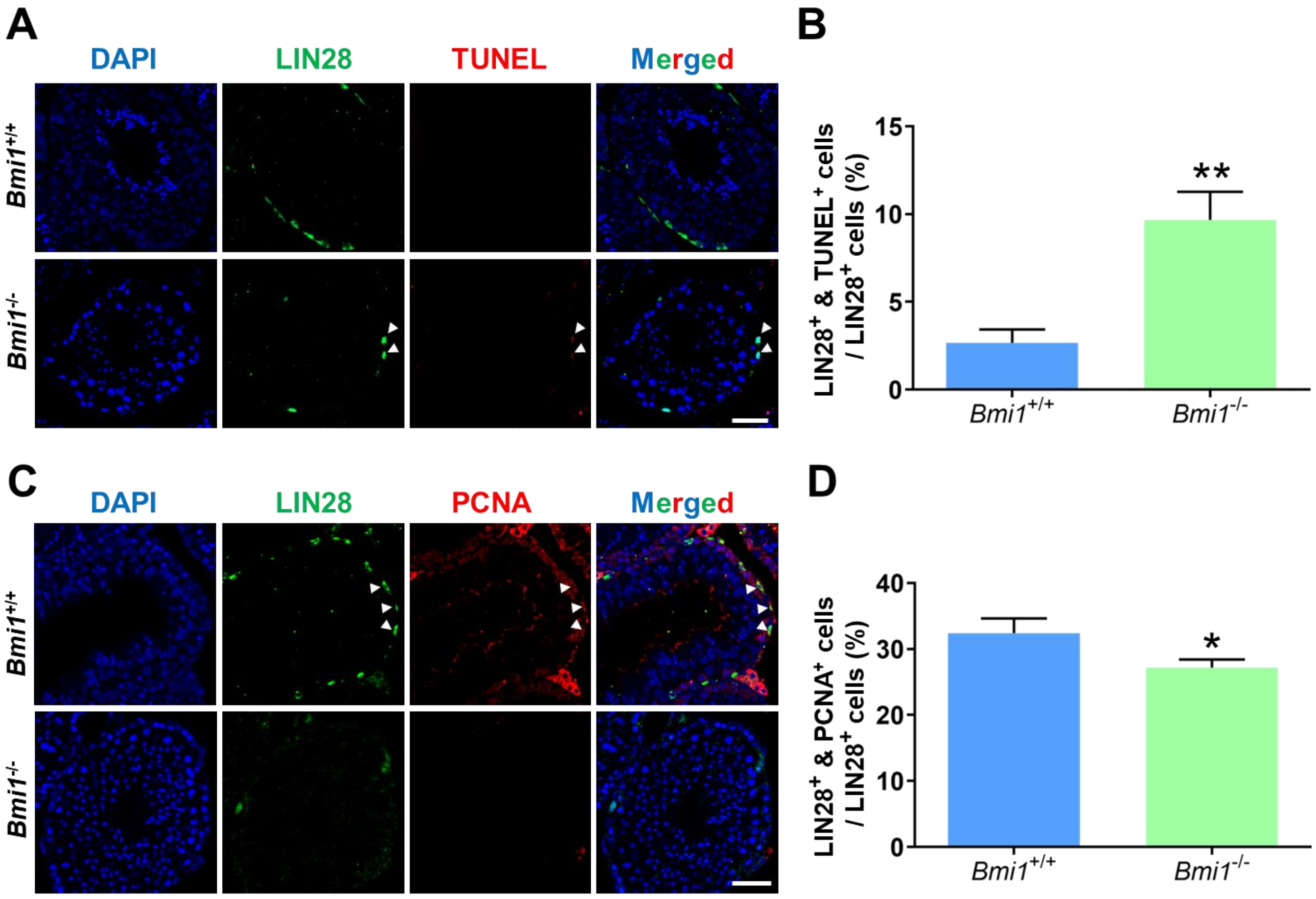

Figure S5. Effects of *Bmi1* knock-out on cell proliferation and apoptosis of SSCs in murine testes. (A) Co-immunostaining for LIN28 and TUNEL in *Bmi1*$^{+/+}$ and *Bmi1*$^{-/-}$ murine testes. **(B)** Quantification of (A). Apoptotic SSCs were indicated with arrowheads. **(C)** Co-immunostaining for LIN28 and PCNA, a marker of proliferative cells, in *Bmi1*$^{+/+}$ and *Bmi1*$^{-/-}$ murine testes. **(D)** Quantification of (C). Proliferative SSCs were indicated with arrowheads. For (A) to (D), data represent mean ± SD, n = 3 independently differentiated groups. Statistical analysis was performed using Student's two tailed t test. **P* < 0.05; ***P* < 0.01. Scale bar, 50 μm.
